# Hydrolysis of Hyaluronic Acid in Lymphedematous Tissue Alleviates Fibrogenesis via T_H_1 Cell-Mediated Cytokine Expression

**DOI:** 10.1038/s41598-017-00085-z

**Published:** 2017-02-24

**Authors:** Sungrae Cho, Kangsan Roh, Jaehyun Park, Yong Seok Park, Minji Lee, Seungchan Cho, Eui-Joon Kil, Mun-Ju Cho, Jeong Su Oh, Hee-Seong Byun, Sang-Ho Cho, Kyewon Park, Hee Kang, Jinmo Koo, Chang-Hwan Yeom, Sukchan Lee

**Affiliations:** 1 Department of Genetic Engineering, Sungkyunkwan University, Suwon, 16419 Republic of Korea; 2 Department of Food Science and Biotechnology, Sungkyunkwan University, Suwon, 16419 Republic of Korea; 3 Department of Oriental Medical Science, Graduate School of East-West Medical Science, Kyunghee University, Yongin, 17104 Republic of Korea; 4 Natural Products Research Department, Gyeonggi Institute of Science & Technology, Suwon, 16229 Republic of Korea; 5 U-Cell Clinic, Seoul, 06605 Republic of Korea

## Abstract

Although surgery and radiation are beneficial for treating cancer, they can also lead to malfunctions of the lymphatic system such as secondary lymphedema. This abnormality of the lymphatic system is characterized by severe swelling, adipogenesis, inflammation, and fibrosis in the lymphedematous region. Moreover, the proliferation of fibrotic tissue in the lymphedematous region generates edema that is no longer spontaneously reversible. No treatment for fibrosis has been validated in patients with lymphedema. In our efforts to develop a therapeutic agent for lymphedema fibrosis, we used a newly established mouse hind limb model. Previous studies have demonstrated that hyaluronic acid accumulates in the lymphedematous region. Thus, we challenged mice with of hyaluronidase (HYAL), with the aim of reducing fibrogenesis. After subcutaneous injections in the lymphedematous mouse leg every two days, the volume of lymphedema had reduced significantly by 7 days post-operation. Histochemical analysis indicated that collagen accumulation and myofibroblast differentiation were decreased in epidermal tissues after HYAL injection. Moreover, it was associated with upregulation of interferon-gamma, increased numbers of Th1 cells, and downregulation of interleukin-4 and interleukin-6 in the lymphedematous region and spleen. These results indicate that hydrolysis of hyaluronic acid can boost an anti-fibrotic immune response in the mouse lymphedema model.

## Introduction

The most widely applied cancer therapies are combinations of surgical therapy, chemotherapy, and radiation therapy. Although these therapies can lead to positive therapeutic results, they can also cause severe side effects, such as chronic lymphedema^1^. For instance, up to 40% of all patients with breast cancer have been estimated to develop lymphedema after therapy^2^. Although lymphedema is not regarded as a life-threatening disease^3^, the quality of life in patients with this disease is significantly affected. Bulky edema in particular has been associated with anxiety, depression, impairment of social relationships, and decreased physical activity^4^. This disease is often caused by cancer therapy, where protein accumulation in the interstitial fluid and lymphatic stasis causes malfunction of the lymphatic system, followed by the development of fibrosis^5^. Moreover, fibrosis is believed to be a key event in secondary lymphedema development^6^.

Fibrosis is part of a two-stage repair process in which connective tissue replaces normal parenchymal tissue via fibroblast proliferation and activation^7^. Fibroblast activation is characterized by apoptosis, resistance to the overproduction of connective tissue matrix, and an increase in the number of myofibroblasts (which are differentiated from fibroblasts)^8^. Fundamentally, these fibroblasts are regulated by T-helper 1 (T_H_1) and T-helper 2 (T_H_2) cells via various cytokines. Most T_H_2 cytokines develop and intensify fibrosis, whereas T_H_1 cytokines stimulate the wound healing response pathway, which counteracts fibrosis^7^.

The most devastating aspect of lymphedema is that the swelling occurs progressively in a delayed manner after surgery. In one previous clinical study, accumulation of TGF-β^+^, a known marker of fibrosis, was observed in patients with lymphedema; collagen was also observed^9^. The importance of fibrosis in lymphedema has also been previously demonstrated. In one study, neutralizing antibodies against interleukin-4 (IL-4) and interleukin-13 (IL-13) were used to treat lymphedema in a mouse model. By neutralizing T_H_2 cytokines, induction of lymphedema in the tail was inhibited^10^. Hyaluronic acid (HA) has also been shown to accumulate in lymphedematous tissue and has been identified as an important marker of lymphedema. Previous studies have also found that HA plays an important role in tissue hydrodynamics, cell movement and proliferation, and participates in a number of cell surface receptor interactions^11^. The primary HA receptor is CD44, which has been reported as a marker of cell activation in lymphocytes^12, 13^. CD44 also participates in T cell activation and T-helper 1(T_H_1) – T_H_2 cell differentiation. Moreover, knockout of CD44 in T cells has been shown to enhance T_H_2 cell differentiation^14^.

Recently, various fragments of hyaluronic acid (as opposed to native high-molecular weight hyaluronic acid) were shown to induce distinct cellular responses, e.g. inflammatory responses, in macrophages and dendritic cells in tissue injury and skin transplant^15, 16^. In addition, HA fragments produced by hyaluronidases can also promote angiogenesis^17^ and hypoxia^18^. Although the various HA fragments are not well characterized, it is known that fragments of various sizes can be produced through a synthesis-degradation balance executed by three types of hyaluronan synthases (HAS) and seven types of hyaluronidases, respectively.

The different sizes of HA fragments have also been shown to have different immunological functions and to act as signaling molecules. For example, the 4-mer hyaluronan has been shown to upregulate the expression of FAS, IL-12, and TNF-α^19–23^. Moreover, fragmentation of HA can affect the wound healing response of fibroblasts^24^. Taken together, the degradation products of HA trigger the expression of IFN-γ, IL-12, and other chemokines that can enhance T_H_1 differentiation^25–27^. Another study found that knockout of the hyaluronan receptor CD44 was associated with increased T cell differentiation to T_H_2 cells and that CD44-knockout splenocytes exhibited lower interferon-gamma expression than wild-type splenocytes^28^.

Since fibrosis is an important factor in lymphedema and severe accumulation of HA has been observed in lymphedematous regions, we treated mice with lymphedema with hyaluronidase to degrade HA. We hypothesized that this treatment would alleviate lymphedema by decreasing fibrogenesis, promoting wound healing, and inducing cytokine expression changes.

This study is part of our work to develop a novel lymphedema therapy without the drawbacks associated with gene therapy and lymph node transplantation^29–31^. We utilized a newly developed hind limb mouse lymphedema model that exhibits symptoms similar to those seen in patients with lymphedema. Using this model, we tested our hypothesis that hyaluronidase (administered via injection) is a potential therapeutic strategy for inhibiting or alleviating fibrogenesis. In this study, we demonstrated that hyaluronidase treatment could inhibit fibrogenesis on a lymphedema mouse model by up-regulation of T_H_1 response and down-regulation of T_H_2 response in lymphedematous tissues. Therefore this observation of hyaluronidase challenging on lymphedema model could be considerable for a clinical approach to alleviate fibrosis on lymphedema patients.

## Materials and Methods

### Development of a novel mouse model of lower limb lymphedema

To develop a novel mouse model of lymphedema and investigate fibrosis caused on lymphedema mouse model, a surgical operation was used to induce lymphedema in the right lower limb of 8-week-old male mice (33–35 g, Charles River Laboratories, Wilmington, MA, USA). All surgical operations were performed under anesthesia [intraperitoneal injections, 33.6 μl of Zoletil 50 (0.6 mg/kg), 22.4 μl of Rompun (0.4 mg/kg), and 144 μl of PBS for each mouse]. Lymphatic system components (lymph nodes and lymphatic vessels) were stained with 0.3% methylene blue (in PBS) on the top of the right foot of each mouse. Incised skin of mouse leg and specific lymphatic components were targeted to effectively induce lymphedema with minimum tissue damage. The components targeted included the superficial inguinal lymph node, the popliteal lymph node, the deep inguinal lymph node, and the femoral lymphatic vessel (SPDF removal model). For complete blockage of lymphatic fluid, we used microforceps that were limited for incision and electro-cauterization for vein suture. After removing lymph node and lymphatic vessels with microforceps and cautery, the incised skins were stitched up with a needle and threads (Kangsan Roh, unpublished master’s thesis, Sungkyunkwan University, 2013). To investigate fibrogenesis and the alleviate effects by hyaluronidase injection on lymphedema mouse models, we sacrificed the mice on 3 day and 7 day after surgery operation for western blotting, histological analysis, quantitative Real-Time PCR, ELISA assay and flow-cytometry analysis. This study was reviewed and approved by the Institutional Animal Care and Use Committee of the Sungkyunkwan University School of Medicine (SUSM). The SUSM facilities are accredited by the Association for Assessment and Accreditation of Laboratory Animal Care International (AAALAC International) and all experimental procedures performed here were in accordance with the guidelines of the Institute of Laboratory Animal Resources (ILAR). This study was also approved by the Administrative Panel of the Laboratory Animal Research Center of Sungkyunkwan University (Approval Number: 12–37).

### Hyaluronidase injection

1500 IU of hyaluronidase was dissolved in 100 μl of phosphate-buffered saline and subcutaneously injected into the leg of each mouse with lymphedema. Injections were performed intraperitoneally under anesthesia 16.8 μl of Zoletil 50 (0.3 mg/kg), 11.2 μl of Rompun (0.2 mg/kg), and 172 μl of PBS for each mouse.

### Masson’s trichrome staining and histological analysis

Mouse legs were fixed in 4% paraformaldehyde solution and incubated in decalcifying solution (Sigma, St. Louis, MO, USA) for 3 days. The legs were then embedded in paraffin and sliced into 3-μm sections for staining. The sections were deparaffinized with o-xylene for 30 min and then rehydrated with 100%, 80%, 70% and 60% (10 min for each step). After rinsing with tap water, nuclei were stained with iron hematoxylin solution for 5 min and then washed with distilled water. Biebrich scarlet-acid fuchsin solution (Sigma) was used to visualize muscle fibers by staining tissue for 10 min. Collagen was then differentiated by staining with 5% phosphomolybdic-phosphotungstic acid solution, after which the sections were rinsed with distilled water. Collagen fibers were stained with 2.5% aniline blue solution for 10 min. After a final wash with distilled water, the slides were dehydrated with 95% ethyl alcohol and cleared in o-xylene. Stained tissue slides were imaged on a Pannoramic MIDI slide scanner (3DHISTECH Ltd, Budapest, Hungary). Manual shots were operated, magnified (40x), and observed under polarized light. Images were analyzed using ImageJ software (NIH, Bethesda, MD, USA; http://rsbwed.nih.gov/ij/). The total blue area was determined as the pixel percentage of the image. Dermal thickness was measured by Pannoramic Viewer version 1.15.2 (3DHISTECH Ltd).

### Immunohistochemistry

Mouse leg tissue slides were deparaffinized and rehydrated as above, with the exception that slides were incubated with peroxidase blocking solution for 30 min at room temperature. After washing in distilled water, antigen retrieval was performed by treating slides with citrate buffer at 90 °C for 20 min. Nonspecific binding sites were then blocked by incubation in blocking solution at room temperature for 10 min. After washing with phosphate-buffered saline, anti-α-SMA (1:300; NB300-978; Novus Biologicals, Littleton, CO, USA), anti-CTGF (1:200; NB100-724; Novus Biologicals), anti-VEGFR-3 (1:200; NBP1-43259; Novus Biologicals) and anti-LYVE-1 (1:200; ab14917, Abcam, Cambridge, MA, USA) antibodies were diluted in dilution buffer. The slides were then incubated with the antibody solutions at 4 °C in a humidified chamber overnight. After washing with phosphate-buffered saline-0.10% Tween 20, the slides were incubated with biotinylated anti-mouse, -rat, -rabbit, -chicken, -guinea pig, -goat, and -sheep IgG antibodies, as appropriate (Gentaur, Brussels, Belgium) for 2 h at room temperature. Anti-goat Alexa 405 (1:500; ab175664, Abcam), anti-rat Alexa 488 (1:300; ab150157, Abcam), and anti-rabbit TRITC (1:300; ab6718, Abcam) secondary antibodies were used according to the manufacturer’s instructions. The fluorescence was observed on a Nikon Ti fluorescence microscope under 40x magnification and the images were analyzed using ImageJ software (NIH, Bethesda, MD, USA; http://rsbwed.nih.gov/ij/). The area, which was stained with anti-α-SMA antibody, was determined as the pixel percentage of the image. The slides scanned using a Pannoramic MIDI slide scanner (3DHISTECH Ltd).

### Quantitative Real-Time PCR

Total RNA was isolated from frozen leg and spleen tissue using TRI reagent (MRC; Molecular Research Center, Cincinnati, OH, USA) according to the manufacturer’s instructions. RNA concentrations were determined using a spectrophotometer to read the absorbance at 260 nm. cDNA was synthesized using MML-V reverse transcriptase (Bioneer Co, Daejeon, Republic of Korea) according to the manufacturer’s protocol. Quantitative real-time PCR was performed using SYBR Premix Ex Taq (TaKaRa, Otsu, Shinga, Japan) and a Rotor-Gene Q system (Qiagen, Chadstone, Victoria, Australia). Data were analyzed using Rotor-Gene Q series software version 2.3.1 (Qiagen). The following genes were amplified with the indicated primers: HAS1 (forward 5′-CACCATCTCAGCCTACCAAGA-3′; reverse 5′-ATCGGCGAAGACTTCTCGGA-3′); HAS2 (forward 5′-TGAACAAAACGGTAGCACTCTG-3′; reverse 5′-ACTTTAATCCCAGGGTAGGTCAG-3′); HAS3 (forward 5′-GATGTCCAAATCCTCAACAAG-3′; reverse 5′-CCCACTAATACATTGCACAC-3′); MMP3 (forward 5′-TCCCGTTTCCATCTCTCTCAAGA-3′; reverse 5′-GGGTACCACGAGGACATCAG-3′); MMP9 (forward 5′-GCGTCGTGATCCCCACTTAC-3′; reverse 5′-CAGGCCGAATAGGAGCGTC-3′);; VEGF-D (forward 5′-GCGACGGTATTCTGTAAAGTGG-3′; reverse 5′-GGACAGGGCTTTGGCAGTTG-3′); fibronectin (forward 5′-GCGACGGTATTCTGTAAAGTGC-3′; reverse 5′-GGACAGGGCTTTGGCAGTTG-3′); IL-6 (forward 5′-TGCAAGAGACTTCCATCCAG-3′; reverse 5′-AGTGGTATAGACAGGTCTGTTGG-3′); CD44 (forward 5′-GACCGGTTACCATAACTATTGTC-3′; reverse 5′-CATCGATGTCTTCTTGGTGTG-3′); and HABP2 (forward 5′-CACCCCTACTACCGCTGTG-3′; reverse 5′-GGTAAACCTGGATCTCCGTCT-3′); α-SMA (forward 5′-CTGACAGAGGCACCACTGAA-3′; reverse 5′-CATCTCCAGAGTCCAGCACA-3′); GAPDH (forward 5′-TGGCAAAGTGGAGATTGTTGCC-3′; reverse 5′-AAGATGGTGATGGGCTTCCCG-3′) was amplified as an internal control.

### Flow Cytometry

Splenocytes were isolated from mice as previously described^32^. Briefly, RBCs were treated with lysis solution for 15 min at room temperature, after which 1 × 10^6^ cells were collected for analysis. For T cell activation, cells were incubated at 37 °C in RPMI1640 medium (GIBCO, Grand Island, NY, USA) supplemented with a cell activation cocktail containing brefeldin A (423303; Biolegend, San Diego, CA, USA) according to the manufacturer’s protocol. The activated cells were washed in phosphate-buffered saline (PBS) and incubated in 5% BSA/PBS for 15 min on ice. Immunostaining was then performed using anti-CD4 Alexa Fluor 488-conjugated antibodies (1:500; 100425; Biolegend). After 3 washes with ice-cold staining buffer, cells were resuspended in fixation buffer (420801; Biolegend), incubated for 15 min on ice, and washed. After permeabilization, cells were immunostained with PE/Cy7 anti-IFN-r (1:500; 505825; Biolegend) and PE/Cy7 anti-IL-4 (1:500; 504117; Biolegend) antibodies. Splenocyte staining was analyzed on a Guava EasyCyte mini instrument and data were analyzed using Cytosoft software version 4.2.1 (Merck Millipore, Billerica, MA, USA).

### Western blotting

Proteins were extracted from frozen tissue with a PRO-PREP protein extraction kit following to the manufacturer’s instructions (iNtRON, Seongnam, Republic of Korea). Protein concentrations were measured using the Bradford assay (Bio-RAD, Munich, Germany). Twenty μg of protein was denatured in sample buffer for 6 min at 95 °C. The samples were loaded on 12% SDS-polyacrylamide gels and transferred to nitrocellulose blotting membranes. The membranes were then blocked with 5% skim milk in Tris-buffered saline at room temperature for 30 min. After 3 washes in Tris-buffered saline-0.10% Tween 20, the membranes were incubated with anti-VEGFR-3 (1:2500; Novus Biologicals), anti-LYVE1 (1:5000; Abcam), anti-CD44 (1:4000; ab24504; Abcam), anti-IFN-γ (1:2000; 505705, Biolegend), and anti-GAPDH (1:5000; sc-25778; Santa Cruz Biotechnology) antibodies at 4 °C overnight. After 4 washes in Tris-buffered saline-0.10% Tween 20 for 20 min, the membranes were incubated with secondary anti-rabbit, anti-rat, or anti-goat antibodies for 1 h at room temperature. After additional washing, immunoreactive bands were detected with ECL substrate (Pierce, Rockford, IL, USA) and exposure to X-ray film (Agfa-Gevaert N.V, Septestraat, Mortsel, Belgium).

### Immunoassays

Protein extracts were generated and their protein concentrations determined as described above. For ELISAs, 20 μg of total spleen and leg protein was coated on 96-well plates in carbonate coating buffer at 4 °C overnight. After 5 washes with phosphate-buffered saline-0.10% Tween 20, the plates were incubated with anti-TGF-β (1:200; ab66043; Abcam), anti-IL-4 (1:500; 504107; Biolegend), anti-IL-12 (1:500; 505207; Biolegend), and anti-IFN-γ (1:500; 505705, Biolegend) antibodies at 4 °C overnight. After an additional 5 washes, the plates were incubated with HRP-conjugated anti-rabbit (1:1000; ADI-SAB-300-J; Enzo Life Sciences, Farmingdale, NY, USA) or anti-rat (1:1000; sc-2006; Santa Cruz Biotechnology) antibodies at room temperature for 4 h. After 5 washes, 100 μl of TMB solution was added and the reaction was allowed to proceed. The reaction was stopped by the addition of sulfuric acid after 40 sec. Optical intensity was measured at 405 nm on an ELISA reader (Tecan Sunrise, Tecan, Switzerland).

## Results

### A new mouse model of leg lymphedema exhibiting collagen accumulation and fibrogenesis in lymphedematous tissues

**Figure 1:**
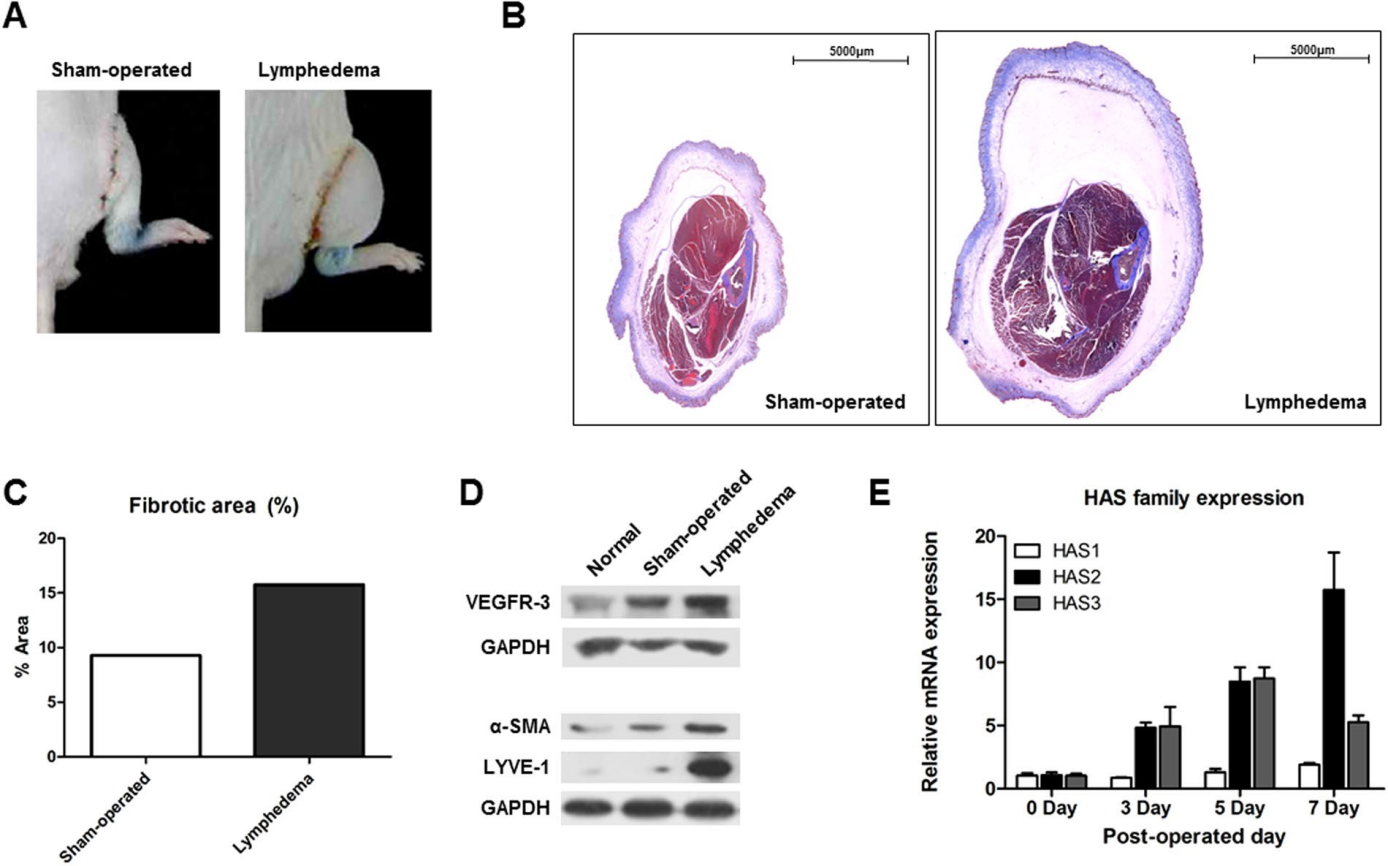
Analysis of the mouse model of lymphedema. Images of the right mouse leg at 3 days postsurgery. (**A**) Sham-operated mouse (without lymphatic damage) and mouse with lymphedema. (**B,C**) Cross-sectional mouse leg histology. Proximal slides were visualized via Masson’s trichrome staining. Scale bar = 5000 μm. Collagen is denoted by blue staining. Percentages of fibrotic areas (blue areas) were determined using Image J software. (**D**) Western blot analysis of the lymphedema markers VEGFR-3 and LYVE-1, alpha-smooth muscle actin, and GAPDH as a loading control. (**E**) Quantitative real-time PCR analysis of the expression of various hyaluronic acid synthases.

The mice with hind limb lymphedema exhibited severe edema on the surgically operated leg at 3 days post-operation (Fig. 1A). Sham-operated (without lymphatic damage) and lymphedema mouse were sacrificed and thin sectioned slides of leg tissues were analyzed with Masson’s tri-chrome staining (Fig. 1B). The blue-colored fibrotic area of Fig. 1B showed that fibrotic area of lymphedema mouse had 21% more than sham-operated mouse (Fig. 1C). The edema volume increased after the operation, reaching a volume about 3 times larger than that in the control mice at 3 days post-operation in the mice with lymphedema. Western blot analysis revealed that two molecular markers of lymphedema vascular endothelial growth factor receptor 3 (VEGFR-3) and lymphatic vessel endothelial hyaluronan receptor 1 (LYVE-1) were upregulated in the mice with lymphedema compared with the control and sham-operated mice. Moreover, the known fibrotic response molecule alpha-smooth muscle actin (α-SMA), which is also a myofibroblast product, was upregulated in the mice with lymphedema (Fig. 1D).

Next, the expression levels of three hyaluronic acid synthase (HAS) genes were investigated by quantitative real-time polymerase chain reaction (qRT-PCR) to extrapolate the potential accumulation of hyaluronic acid in lymphedematous tissues. The kinetics of the expression levels were analyzed over time after the operation and all expression ratios were normalized to their day 0 values. This analysis revealed that all three HAS genes exhibited gradual postoperative increases in expression in the mice with lymphedema. Moreover, HAS2, whose product is involved in synthesis of high molecular weight hyaluronic acid (HMWHA), was more significantly increased on day 7 post-operation compared to HAS1 and HAS3 (Fig. 1E).

### Subcutaneous injection of hyaluronidase inhibits fibrogenesis on the dermis layer of the lymphedematous leg

**Figure 2:**
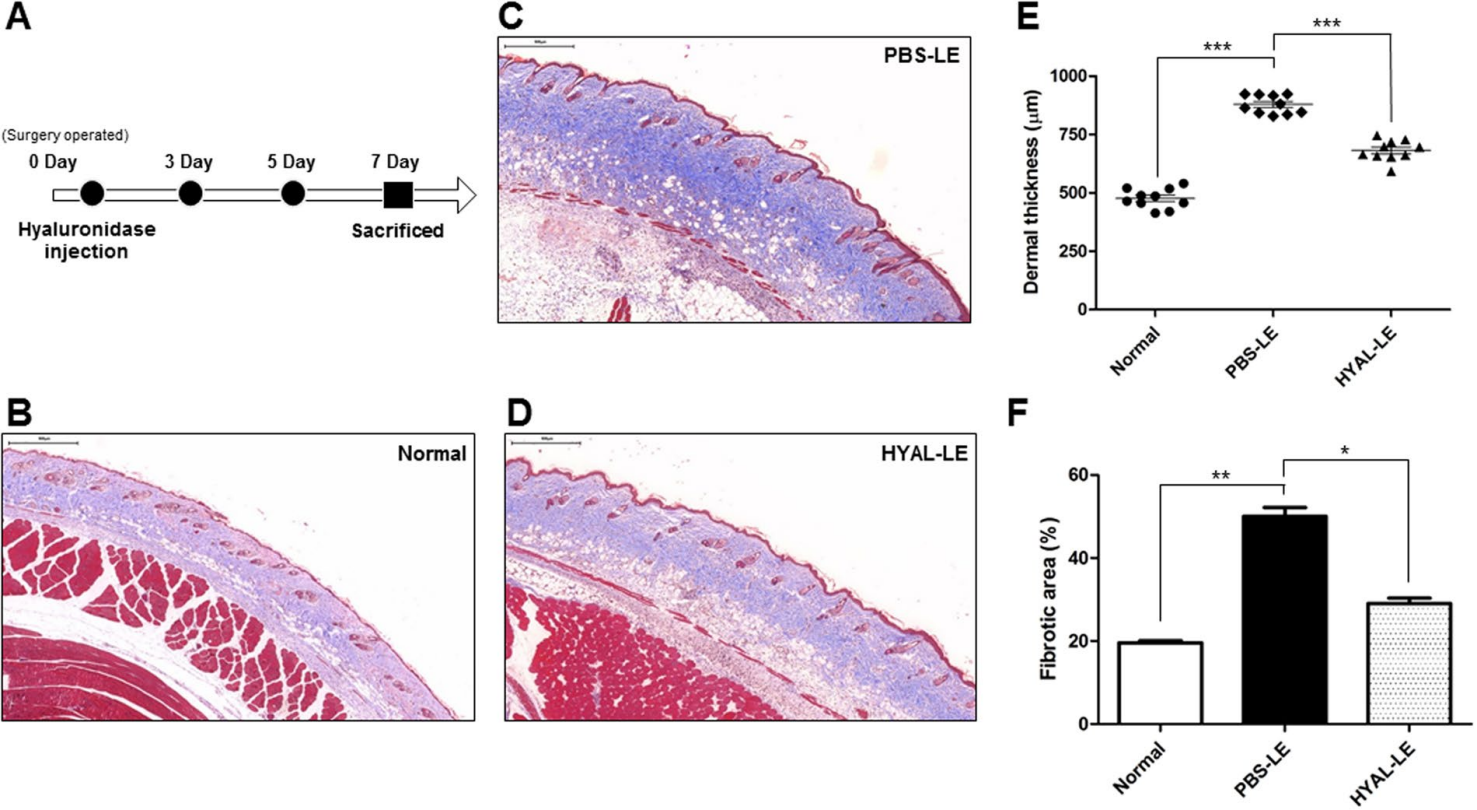
Subcutaneous injection of hyaluronidase alleviates lymphedema in a mouse model. At 7 days postsurgery, PBS-injected mice with lymphedema (PBS-LE) and hyaluronidase-injected mice with lymphedema (HYAL-LE) were sacrificed. Cross-sections of the legs with edema were then analyzed. (**A,B**,**C**) Representative Masson’s trichrome staining of fibrotic areas. Scale bar = 500 μm. (**D,E**) Image J analysis of dermal thickness and fibrotic (blue) areas. Data are presented as means ± SEMs. *P < 0.05, **P < 0.005, ***P < 0.001. (**F**) Schematic diagram of the PBS and hyaluronidase injection schedule. •, hyaluronidase and PBS injection; ■, time of sacrifice.

**Figure 3:**
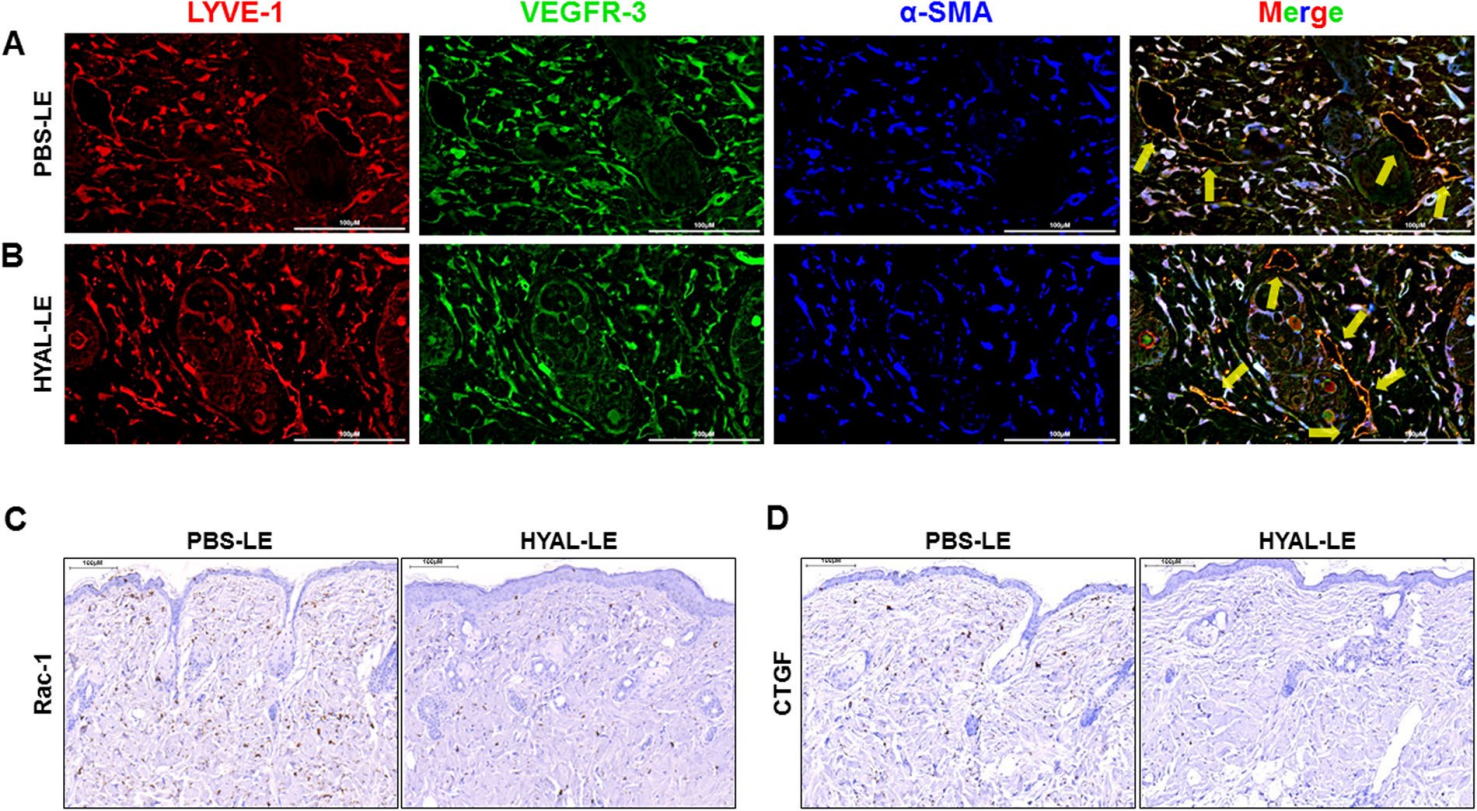
Hyaluronidase treatment restores dilated lymphatic vessels to round lymphatic vessels and downregulates the expression of fibrosis markers. Histological analysis of the effects of hyaluronidase on fibrosis in lymphedema. (**A,B**) Immunofluorescence staining of LYVE-1, VEGFR-3, and alpha-smooth muscle actin in proximal mouse leg sections. Scale bar = 100 μm. Regions of LTVE-1 and VEGFR-3 colocalization in the lymphatic vessels are marked with yellow arrows. (**C,D**) Immunohistochemistry analysis of RAC1 and CTGF in the dermis layer of the mouse leg. Scale bar = 500 μm.

After the hyaluronidase injections (Fig. 2A), the normal mice, PBS-injected mice with lymphedema (PBS-LE), and hyaluronidase-injected mice with lymphedema (HYAL-LE) were sacrificed and tissue cross-sections were subjected to histological analysis. Collagen accumulation in lymphedematous tissues, which is related to fibrosis, was analyzed by Masson’s trichrome staining, which yields a blue color (Fig. 2B,C and D). The PBS-LE mice showed more intense blue staining in a larger area of lymphedematous tissue than the HYAL-LE mice (Fig. 2C,D). However, subcutaneous injection of hyaluronidase into the lymphedematous leg reduced the extent of collagen accumulation in the dermal layers, as demonstrated by the decreased dermal thickness and fibrotic area. These histochemical results were analyzed quantitatively using two different programs. Specifically, the width of the skin layer to the muscularis mucosae was quantitatively analyzed with Pannoramic Viewer (Fig. 2E) and color threshold and particle analysis was performed using Image J (Fig. 2F). To investigate the ability of HYAL to reduce fibrogenesis on the molecular level, the expression levels and localizations of the well-known lymphangiogenesis markers LYVE-1, VEGFR-3, and α-SMA in the lymphedematous tissues were analyzed by fluorescence microscopy (Fig. 3A,B). Co-staining of LYVE-1 and VEGFR-3 revealed regeneration of lymphatic vessel microarchitecture. In the PBS-LE mice, the lymphatic vessels were dilated and strong anti-α–SMA staining was observed around the lymphatic vessels. In contrast, the ellipse shapes of the lymphatic vessels were observed and these shapes were not observed in the HYAL-LE mice. When myofibroblast cells were stained with anti-α–SMA antibody and the areas were measured with ImageJ software, the blue area diagnosed as myofibroblast cells was 13.2% in PBS-LE and 8.43% in HYAL-LE. To determine whether the therapeutic effects of HYAL involved inhibition of fibroblast proliferation and differentiation to myofibroblasts, we performed immunohistochemical staining of anti-Ras-related C3 botulinum toxin substrate1 (Rac1) and anti-connective tissue growth factor (CTGF) (Fig. 3C,D). Rac1 and CTGF both exhibited decreased expression in the HYAL-LE mice; however, no changes were observed in the PBS-LE mice. Taken together, our histological data indicate that subcutaneous injection of hyaluronidase into the lymphedematous tissues suppresses fibrogenesis. Moreover, these preventive effects are mediated by inhibition of myofibroblast differentiation and the rescue of impaired lymphatic vessels.

### Investigation of the hyaluronidase-induced wound healing response and the expression of hyaluronan-binding protein 2

**Figure 4:**
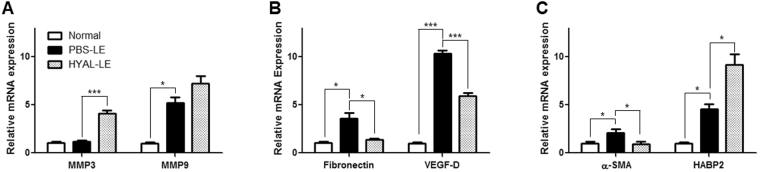
Hyaluronidase injection induces anti-fibrotic responses. Molecular analysis of mouse leg lysates. (**A–C**) Quantitative real-time PCR analysis of VEGF-D, fibronectin, MMP3, MMP9, α-SMA and HABP2. (**A**) Increased expression levels of MMP3 and MMP9 were observed, (**B**) Whereas decreased expression levels of VEGF-D, and fibronectin were observed in the hyaluronidase-injected mice. (**C**) The expression of α-SMA was decreased with hyaluronidase treatment, but HABP2 expression was increased. Data are presented as means ± SEMs. *P < 0.05, ***P < 0.001.

Since our data indicated that hyaluronidase injection inhibited dermal fibrogenesis in mice with lymphedema, we performed quantitative real-time polymerase chain reaction analysis to further investigate the preventive effects of HYAL on fibrogenesis. First, the expression of matrix metalloproteinase (MMP) was analyzed to determine if HYAL injection inhibits collagen synthesis and stimulates collagen degradation. The expression levels of MMP3 and MMP9, two enzymes that degrade the extracellular matrix, were both increased by HYAL injection (4-fold and 1.3-fold, respectively) (Fig. 4A). To evaluate the extent of the preventive effect of HYAL on fibrogenesis, the mRNA expression levels of vascular endothelial growth factor D (VEGF-D) and fibronectin were also investigated (Fig. 4B). VEGF-D expression was 0.4-fold lower in the HYAL-LE mice compared to the PBS-LE mice; similarly, fibronectin expression was downregulated 0.3-fold in the HYAL-LE mice compared to the PBS-LE mice. In addition, α-SMA, a major marker of fibrosis, was decreased in the HYAL-LE mice. Moreover, the expression of the anti-fibrotic enzyme hyaluronan binding protein 2 (HABP2) was increased in the HYAL-LE mice compared to the PBS-LE mice (Fig. 4C). Together, these results indicate that HYAL injection induced the expression of matrix degradation proteins and downregulated the expression of an inhibitor of matrix degradation, which is also a known marker of fibrosis. Moreover, the upregulation of HABP2 implies that more low-molecular weight hyaluronic acid (LMWHA) had accumulated in the HYAL-LE group than in the PBS-LE group.

### Upregulation of T_H_1-driven cytokines in the spleen of hyaluronidase-treated mice

**Figure 5:**
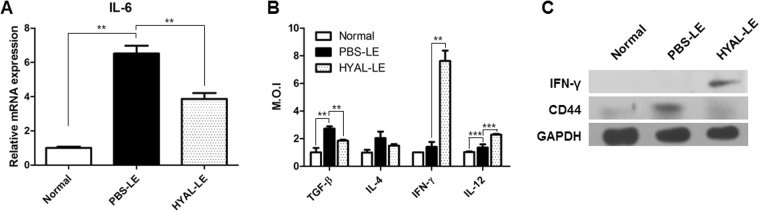
Subcutaneous hyaluronidase injection alters cytokine expression. Analysis of cytokine expression in total mouse leg tissue. (**A**) Quantitative real-time PCR analysis of IL-6 mRNA expression. Data are presented as means ± SEMs. (**B**) ELISA analysis of the levels of TGF-β, IL-4, IFN-γ, and IL-12. Data are expressed as median optical intensities. Data are presented as means ± SEMs. (**C**) Representative western blot of IFN-γ and CD44 expression. **P < 0.01, ***P < 0.001.

Anti-fibrotic responses such as upregulation of MMPs and downregulation of VEGF-D, and fibronectin are known to be mediated by T cell driven cytokines. To investigate T cell mediated anti-fibrotic responses, we analyzed the expression of interleukin-6 (IL-6) by qRT-PCR (Fig. 5A). We also analyzed the expression levels of interleukin-4 (IL-4), interleukin-12 (IL-12), transforming growth factor beta (TGF-β), and interferon gamma (IFN-γ) by ELISA (Fig. 5B). On the mRNA expression level, IL-6 was decreased about 0.3-fold in the HYAL-LE mice compared to the PBS-LE mice. The T_H_2 cell marker and profibrotic cytokine IL-4 was downregulated 0.2-fold in the HYAL-LE mice compared to the PBS-LE mice, as assessed by ELISA (Fig. 5B), although this reduction was not statistically significant. However, the myofibroblast differentiation factor TGF-β was significantly downregulated in HYAL-LE mice compared to PBS-LE mice. In contrast, the anti-fibrotic factor IFN-γ was significantly upregulated (4.0-fold) in HYAL-LE mice compared to PBS-LE mice. Consistent with this finding, IFN-γ was detected by western blot hybridization in lymphedematous tissues from HYAL-LE mice (Fig. 5C). In addition, IL-12 expression was also 1.4-fold higher in HYAL-LE mice compared to PBS-LE mice.

### Subcutaneous injection of hyaluronidase into lymphedematous tissues resulted in enhanced T_H_1 differentiation and altered expression of hyaluronic acid receptors

**Figure 6:**
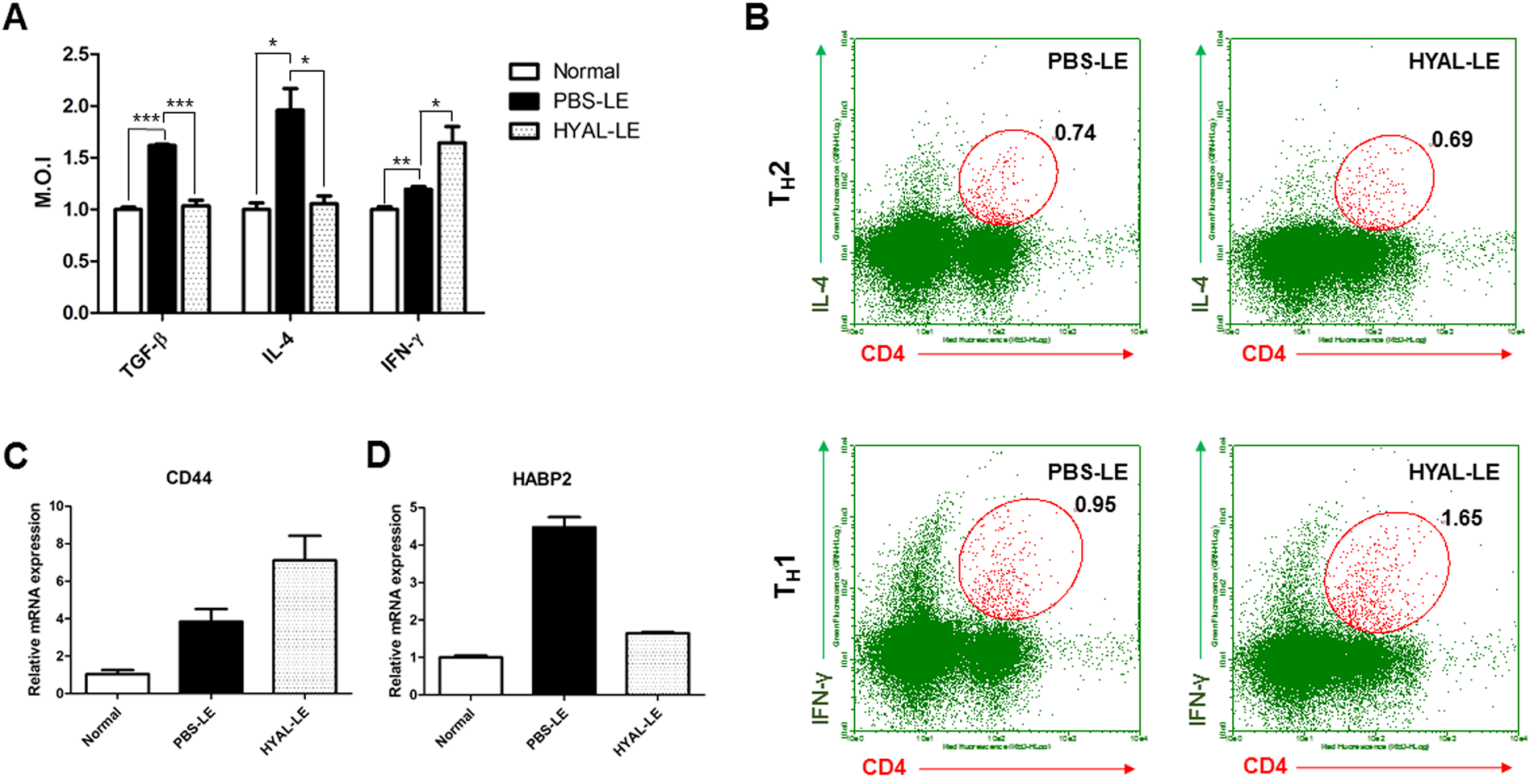
Hyaluronidase injection significantly alters splenocyte T cell populations. Analysis of cytokine expression and T_h_ cell population analysis in total mouse spleen tissue. (**A**) ELISA analysis of the levels of TGF-β, IL-4, IFN-γ on spleen. Data are expressed as median optical intensities. Data are presented as means ± SEMs. (**B**) Representative flow cytometry results of T_H_1 and T_H_2 cell populations. Data are presented as means ± SEMs. (**C**,**D**) Quantitative real-time PCR analysis of IL-6 and HABP2 mRNA expression. Data are presented as means ± SEMs. *P < 0.05, **P < 0.005, ***P < 0.001.

To determine whether the preventive effects of HYAL in lymphedematous tissues were mediated by local or systemic effects, we analyzed the levels of TGF-β, IL-4, and IFN-γ in spleen lysates by ELISA. The expression patterns were consistent with the Western analysis; i.e. TGF-β and IL-4 were downregulated in the HYAL-LE mice (Fig. 6A). However, IFN-γ was more highly expressed in the HYAL-LE mice than in the PBS-LE mice. These results were supported by fluorescence-activated cell sorting (FACS) analysis of spleen cells from HYAL-LE mice (Fig. 6B). In addition to immunostaining for IL-4 and IFN-γ, cells were also co-stained with CD4 for analysis of the T_H_ cell populations. The population of T_H_2 cells was slightly decreased (from 0.74 to 0.69) in cells co-expressing IL-4 and CD4. However, the percentage of T_H_1 cells was increased by 1.7-fold in HYAL-LE mice compared with PBS-LE mice. Taken together, these results indicate that HYAL-mediated inhibition of fibrogenesis is preferentially and systemically mediated by T_H_1 cell differentiation.

### Opposite expression patterns of CD44 and HABP2 in the lymphedematous leg and spleen of HYAL-LE mice with different T_H_ cell populations

Hyaluronidase can degrade high molecular weight hyaluronic acid (HMWHA) into low molecular weight hyaluronic acid (LMWHA). To determine whether LMWHA is a potential signaling molecule, we analyzed the induction patterns of hyaluronic acid receptors in the lymphedematous mouse leg and spleen. The hyaluronic acid binding receptor CD44 exhibited opposite expression patterns in the lymphedematous leg vs the spleen of HYAL-LE mice (Figs 5C and 6C). Specifically, CD44 expression was downregulated in the lymphedematous leg of HYAL-LE mice, whereas it was upregulated in spleen cells from HYAL-LE mice compared with PBS-LE mice. In contrast, HABP2, which is involved in a hyaluronic acid signaling pathway, was downregulated in the spleen (Fig. 6D). Taken together, these results indicate that CD44 and HABP2 exert different functions on lymphedematous tissue vs the spleen. In addition, flow cytometry analysis of mouse splenocytes showed that the T_H_1 population was increased approximately 1.6-fold, whereas the T_H_2 cell population was decreased approximately 0.9-fold in HYAL-LE mice compared with PBS-LE mice.

## Discussion

In our efforts to develop a novel lymphedema therapy, we recently established a novel mouse model of leg lymphedema (Kangsan Roh, unpublished master’s thesis, Sungkyunkwan University, 2013). Several lymphedema animal model systems were conducted with removing lymphatic components for insufficient lymphatic function^33–35^. Also, we established lower limb lymphedema mouse model through removing the superficial inguinal lymph node, the popliteal lymph node, the deep inguinal lymph node, and the femoral lymphatic vessel. With decreased lymphatic function, this mouse model presented tissue swelling and formation of edema occurred as consequences of abnormal lymphatic system and also exhibited typical expression patterns of lymphedema markers such as VEGFR-3, LYVE-1, and α-SMA, in addition to collagen accumulation. Clinical studies have revealed that patients with lymphedema exhibit 8 times more accumulated HA on their arms and legs compared with control patients. Consistent with this finding, here we found that HA accumulated to higher levels in surgically-induced lymphedematous legs compared with normal legs. Some evidence suggest that HA accumulation in lymphedematous tissues is due to the upregulation of hyaluronic acid synthase^36^.

At the 7 day time point after the 3 hyaluronidase injections, the swelling of the lymphedematous tissues in the HYAL-LE group had reduced to the level in the normal group. Moreover, histological analysis revealed that the dermal thickness and fibrotic areas of the HYAL-LE group were reduced significantly and were comparable to those of the PBS-LE group (negative control). Based on these results, we examined the expression of Rac1 and CTGF, markers of proliferation and fibroblast differentiation, and found that they were also reduced after hyaluronidase injection. These results suggest that hyaluronidase injection, which produced HA fragments, also inhibited fibroblast proliferation and differentiation. As shown in Fig. 3A and B, α-SMA co-localized with VEGFR-3 and LYVE-1 and was detected at lower levels in HYAL-LE mice compared with PBS-LE mice. Consistent with the decreased intensity of α-SMA as revealed by immunohistochemical staining, the lymphatic vessels in the HYAL-LE mice exhibited normal morphology. This observation is supported by a previous study that demonstrated the importance of fibrosis in lymphatic regeneration in a mouse model of tail lymphedema^6^. Finally, these results imply that subcutaneous injection of hyaluronidase inhibits fibroblast activation, which affects the recovery of lymphatic vessels. This finding is supported by previous studies demonstrating that TGF-β1 negatively regulates lymphatic regeneration via myofibroblast activation accompanied by fibrosis^37–40^.

Analysis on the molecular level also supported an anti-fibrotic response induced by hyaluronidase injection. Specifically, hyaluronidase injection altered the expression levels of MMPs, VEGF-D, fibronectin, and α-SMA. Downregulation of these markers, along with high expression of HABP2, has been positively correlated with vascular integrity^41^. In contrast, CD44 expression in the leg of hyaluronidase-treated mice was decreased by hyaluronidase treatment (Fig. 5C). Interestingly, CD44 has been shown to mediate fibrocyte invasion and migration in fibroblasts^42^. We hypothesize that the degradation of high molecular weight HA (>1 × 10^6^ Da) led to a reduction in CD44 expression and that vascular permeability was also increased with the change in HABP2 expression. Furthermore, we propose that these changes alleviate fibrogenesis by inhibiting fibroblast invasion and migration, thus decreasing the probability that the immune response is activated. Previous studies have shown that low molecular weight HA (<1 × 10^6^ Da) induces immune and inflammatory responses^43–45^. Therefore, hyaluronidase-mediated degradation of high molecular weight HA could explain these results. Moreover, cytokine analysis of the HYAL-LE mice revealed that anti-fibrotic cytokines (IL-12 and IFN-γ) were systemically induced by hyaluronidase injection (Fig. 5B). However, pro-fibrotic cytokines such as IL-4 and TGF-β were downregulated by hyaluronidase injection. We showed that the expression of IFN-γ was increased after hyaluronidase treatments and T_H_2 cytokines such as TGF-β and IL-4 were decreased. Even though the responses of both T_H_1 and T_H_2 were regulated negatively during lymphatic vessel formation shown by previous researches^46, 47^, we suggested that the alleviation of fibrosis and regeneration of lymphatic vessel were caused by the increased T_H_1 response and the decreased T_H_2 response via the changed balances of T_H_1/T_H_2 based on the observation through this study. These results indicate that the anti-fibrotic effects of hyaluronidase are strongly correlated with the regulation of fibroblast activation and the inhibition of myofibroblast differentiation through HA receptors^48^ and HABP2, also known as factor VII activating protease^49, 50^. Of particular note, the T_H_1 and T_H_2 cell polarization ratio has also been related to fibrosis^7^. FACS analysis of cytokine expression in the spleen and splenocytes provided further support for our findings (Fig. 6B). HA and its receptors play important roles in fibroblast proliferation and protein synthesis, which are important for inducing fibrosis^11^, in addition to important roles in the activation and differentiation of T cells^14, 51^. Moreover, the interactions between HA and its receptors affect cytokine expression in macrophages^52^ and fibrocyte proliferation in response to high molecular weight HA^48^. As shown in Fig. 6B, the breakdown of HA by HYAL induced T_H_1/T_H_2 population changes *in vivo*. We conclude that hyaluronidase exerts systemic and local preventive effects against lymphedema, based on our analyses of T_H_ cells and lymphedematous tissues, respectively. In the context of the T_H_1/T_H_2 paradigm^7^, we found that hyaluronidase treatment increased T_H_1-cell cytokines, suppresses fibrogenesis, and aided wound healing, a finding that is consistent with previous studies^53–57^. Hyaluronidase treatment has also been associated with lymphatic generation^38, 58, 59^. Recent study shows that T_H_2 differentiation was targeted for therapy of lymphedema. It was demonstrated that CD4^+^ T-cell inflammation and T_H_2 differentiation led to lymphatic stasis. Furthermore, inhibition of T_H_2 responses by anti-IL-4 antibody treatment reduced fibrosis on lymphedema and also blockade T_H_2 responses improved lymphatic function, without alteration expression of vascular growth factors^10, 60^. The most important findings of our study are: 1) the decreased expression of T_H_2 cytokines and related molecules such as IL-4 and IL-13; and 2) the reduced population of T_H_2 cells in the HYAL-treated group. Since HA is known to circulate in the body^61^, we hypothesize that local injection of hyaluronidase not only affects fibroblasts and myofibroblasts near the injection site, but also exerts systemic effects on the immune system via HA fragments. Indeed, several reports have indicated that HA fragments can influence immune cells, at least in *in vitro* systems^22, 25, 62^. According to previous studies^63–65^, the HA-HA receptor interaction preferentially mediates T_H_1 differentiation of T cells. In addition, IL-12 and other lymphocyte cytokines have also been associated with T_H_1 differentiation. The upregulation of IL-12 also indicates that population changes in T_H_ cells could be mediated by dendritic cells, which are recognized by HA fragments^26, 66, 67^.

Although it would also be informative to study population changes of other immune cells besides T_H_ cells, such work was outside the scope of the present investigation. To understand the mechanism by which hyaluronidase exerts its preventive effects and to investigate its potential as a therapeutic drug, further studies must focus on the immune responses induced by HA fragments of various sizes and population changes of immune cells in response to these fragments. Moreover, since HA fragments of different sizes have different functions, it will be important to determine the sizes of the HA fragments in lymphedematous tissues produced by hyaluronidase injection. Importantly, no known therapeutic agents are yet available to alleviate lymphedema. Thus, our findings that hyaluronidase is a promising candidate for lymphedema treatment are encouraging and warrant further investigation.

## Electronic supplementary material

Supplementary Figure 1
