# Supplementary material for: Hydrolysis of Hyaluronic Acid in Lymphedematous Tissue Alleviates Fibrogenesis via TH1 Cell-Mediated Cytokine Expression
Source: Sci Rep. 2017 Feb 24;7:35. doi: 10.1038/s41598-017-00085-z (PMC5428353; doi:10.1038/s41598-017-00085-z)
Supplement: Supplementary file 1 — Supplementary Figure 1 [file 41598_2017_85_MOESM1_ESM.pdf]

Hydrolysis of Hyaluronic Acid in Lymphedematous Tissue Alleviates Fibrogenesis via T_H1 Cell-Mediated Cytokine Expression

Sungrae Cho[1], Kangsan Roh[1], Jaehyun Park[1], Yong Seok Park[1], Minji Lee[1], Seungchan Cho[1], Eui-joon Kil[1], Mun-ju Cho[1], Jeong Su Oh[1], Hee-seong Byun[1], Sang-ho Cho[1], Kyewon Park[2], Hee Kang[3], Jinmo Koo[4], Chang-Hwan Yeom[5], Sukchan Lee[1*]

Supplementary Figure 1

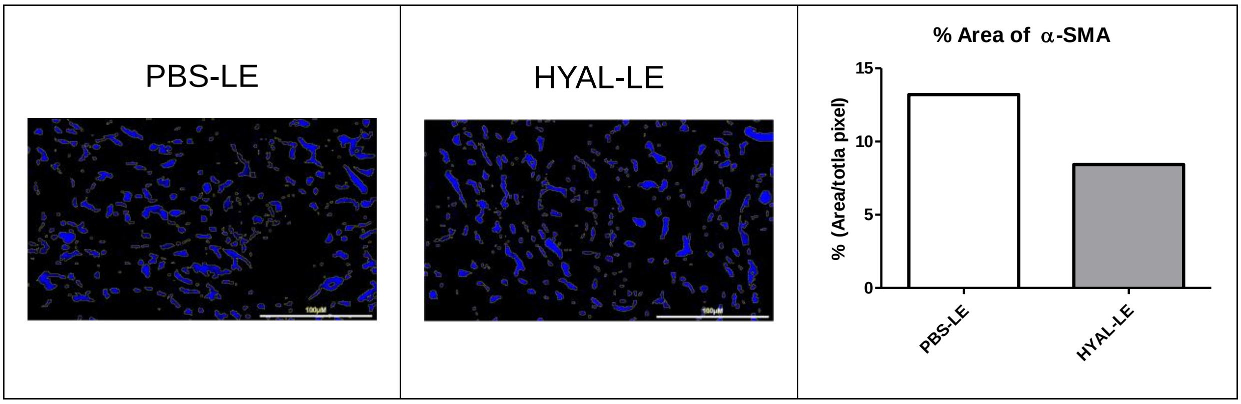

Supplementary Figure 1.

Processed images were used for calculating the area of α-SMA from Figure 3A and 3B. By ImageJ software, same color threshold was applied on PBS-LE and HYAL-LE and the selected blue area was calculated as % of blue-colored area / total area.
